# The distribution of runs of homozygosity and selection signatures in six commercial meat sheep breeds

**DOI:** 10.1371/journal.pone.0176780

**Published:** 2017-05-02

**Authors:** Deirdre C. Purfield, Sinead McParland, Eamon Wall, Donagh P. Berry

**Affiliations:** 1 Animal & Grassland Research and Innovation Center, Teagasc, Moorepark, Fermoy, Co. Cork, Ireland; 2 Sheep Ireland, Bandon, Co. Cork, Ireland; University of Queensland, AUSTRALIA

## Abstract

Domestication and the subsequent selection of animals for either economic or morphological features can leave a variety of imprints on the genome of a population. Genomic regions subjected to high selective pressures often show reduced genetic diversity and frequent runs of homozygosity (ROH). Therefore, the objective of the present study was to use 42,182 autosomal SNPs to identify genomic regions in 3,191 sheep from six commercial breeds subjected to selection pressure and to quantify the genetic diversity within each breed using ROH. In addition, the historical effective population size of each breed was also estimated and, in conjunction with ROH, was used to elucidate the demographic history of the six breeds. ROH were common in the autosomes of animals in the present study, but the observed breed differences in patterns of ROH length and burden suggested differences in breed effective population size and recent management. ROH provided a sufficient predictor of the pedigree inbreeding coefficient, with an estimated correlation between both measures of 0.62. Genomic regions under putative selection were identified using two complementary algorithms; the fixation index and hapFLK. The identified regions under putative selection included candidate genes associated with skin pigmentation, body size and muscle formation; such characteristics are often sought after in modern-day breeding programs. These regions of selection frequently overlapped with high ROH regions both within and across breeds. Multiple yet uncharacterised genes also resided within putative regions of selection. This further substantiates the need for a more comprehensive annotation of the sheep genome as these uncharacterised genes may contribute to traits of interest in the animal sciences. Despite this, the regions identified as under putative selection in the current study provide an insight into the mechanisms leading to breed differentiation and genetic variation in meat production.

## Introduction

Domestication and the subsequent selection of animals for either economic or morphological features can leave a variety of imprints on the genome of a population. This selection, combined with the natural adaptation to local environments, has resulted in over one thousand different sheep breeds that vary phenotypically [1]. Understanding the genetic diversity among these sheep breeds can contribute to the success of many genomic analyses including genomic selection and QTL detection through genome-wide association studies [2, 3].

Genomic regions subjected to selection frequently show signatures such as reduced nucleotide diversity, stretches of homozygous loci (i.e. runs of homozygosity; ROH), shifted site frequency spectrum and reduced recombination rate. The presence of continuous lengths of homozygous genotypes in an animal can be attributed to the inheritance of identical haplotypes from both parents [4]. The extent and frequency of these ROH can inform on both the ancestry of an animal itself, as well as of the population as a whole. Particularly, consanguinity may be indicated from the presence of long ROH; the longer the ROH the more likely that recent inbreeding occurred within a pedigree, as limited opportunity existed for recombination to break up these haplotype segments [4]. As a result, ROH are widely used as a predictor of whole genome inbreeding levels [5–7]. Moreover, as selection is often characterised by local reductions in haplotype diversity, the distribution of ROH patterns across the genome can inform on genomic regions that have potentially been subjected to recent and/or ancient selective pressure [8, 9].

The reduction in the genetic variation surrounding a beneficial mutation is known as a “selective sweep” and occurs due the positive selection pressure altering the frequency of the favourable allele(s) over time [10]. If a population undergoes recent intensive selection pressure, extended linkage disequilibrium (LD) patterns between the mutation and neighbouring SNPs are observed [11, 12]. This often leads to the emergence of different haplotypes in populations that have been subjected to varying selection pressures [13]. Several methods exist to detect regions of selection, and one such commonly used measure is Wright’s fixation index (F_ST_) [14]. The fixation index is a single SNP test that is routinely used to identify highly differentiated alleles that have undergone divergent selection among populations [15–17]. However, one major concern highlighted by Fariello et al., [18] is that the F_ST_ approach assumes that all populations have the same effective population size and were derived independently from the same ancestral population; if this is not the case, false positive F_ST_ signals could be detected. Therefore, Fariello et al., [18] proposed the hapFLK statistic, which is a haplotype-based extension of the FLK statistic developed by Bonhomme et al., [19], that can account for both population structure and haplotype information. In contrast, the FLK test, which is an extension of the Lewontin and Krakauer (LK) test, is a single SNP test that accounts for population size heterogeneity and structure to compute a global F_ST_ for each SNP [19].

Both hapFLK and F_ST_ have been previously applied to varying sheep populations to identify regions of the genome under selection [1, 16, 18, 20–23]. These studies have successfully identified several genomic regions associated with morphological traits, reproductive performance, nematode resistance, body size and skeletal morphology, which have been targeted by both natural and artificial selection during domestication. However, detecting regions of selection associated with quantitative polygenic traits such as growth and muscularity, is hampered by the standing variation existing at many loci in these traits [24]. As a result, selection for quantitative traits is often driven by polygenic adaptation i.e. shifts but not fixation in the allele frequencies of thousands of loci that have small effects on a trait [24, 25]. Therefore, hard selective sweeps are rarely detected for such quantitative traits. The breeds used in the current study, with the exception of the Belclare breed where emphasis remains on prolificacy [26], have all been subjected to selection for meat and growth related traits in recent years. Despite the emphasis on terminal related traits within these breeds, substantial differences in phenotype and morphology exist, and they provide a considerable resource for deciphering the genetic variation that exists between terminally selected breeds.

Therefore, the objective of the present study was to quantify the genetic diversity in six commercial sheep breeds using both ROH and selection signatures with the aim of identifying genomic regions that have been subjected to selection. In addition, the detected ROH will be assessed for their predictive ability of inbreeding through comparison with the traditional pedigree inbreeding coefficient and alternative genomic inbreeding coefficients. Results from this study will be useful in identifying genomic regions that differentiate among breeds and, through their biological annotation, provide insights into the mechanisms underlying past selection practices.

## Methods

Animal Care and Use Committee approval was not obtained for this study because the data were from an existing database.

### Genotypic data

A total of 51,135 biallelic SNPs from the Illumina OvineSNP50 genotype panel were available on 3,289 animals from six breeds. Breeds represented included Belclare (n = 658), Beltex (n = 64), Charollais (n = 665), Suffolk (n = 784), Texel (n = 489) and Vendeen (n = 629). Individuals and SNPs with a call rate <95% were discarded, as were 1,976 non-autosomal SNPs and SNPs with a MAF <0.01 across all individuals. Finally, any SNP that, within breed, deviated (p<0.1x10^-6^) from Hardy-Weinberg equilibrium was discarded. Following all edits, 42,182 autosomal SNPs remained on 3,191 sheep. SNPs were positioned using the sheep (*Ovis aris*) genome assembly 3.1 (OAR 3.1).

### Population structure analyses

To understand population structure within and between breeds principal component analysis (PCA) using EigenStrat [27] and ancestry models implemented in ADMIXTURE 1.2.3 [28] were performed. To ensure uncorrected LD did not distort the results, pairwise SNP pruning was completed using PLINK [29] prior to analyses. This involved removing one locus from each SNP pair where LD (r^2^) exceeded 0.1 within 50-SNP blocks. The cross validation procedure in ADMIXTURE was used to estimate the most likely number of genetic populations (clusters of K) between the breeds, considering values of K from 2 to 8. PCA plots were constructed using the first four components from the analysis.

### Effective population size

The historical and current effective population size of each of the six breeds was estimated using the SNeP tool as described by Barbato et al. [30]. This approach is based on the relationship between the variance in LD between adjacent SNPs and the effective population size in the presence of a mutation to infer ancestral and recent effective population sizes [31];

$$N_{T\left(t\right)}={\left(4f\left(c_{t}\right)\right)}^{-1}\left(E{\left[r_{adj}^{2}|c_{t}\right]}^{-1}-\alpha \right)$$

where *N*_*T(t)*_ is the effective population size *t* generations ago calculated as *t* = (2*f*(*c*_*t*_))^−1^ [20], *c*_*t*_ is the recombination rate for a specific physical distance between SNPs estimated using Sved & Feldman [32], $r_{adj}^{2}$ is the LD value adjusted for sample size and α is a correction for the occurrence of mutations. Only SNPs with a MAF >0.05 were used to estimate the effective population size.

### Runs of homozygosity

Runs of homozygosity were defined in each of the six populations of sheep using a sliding window approach of 50 SNPs in PLINK v1.09 [29], as previously described for cattle by Purfield et al. [5]. A maximum of two SNPs with a missing genotype, and up to one possible heterozygous genotype was permitted per ROH window. To minimize the detection of ROH that could occur by chance, the minimum number of SNPs needed to constitute a ROH (l) was estimated using the method proposed by Lencz et al., [33];

$$l=\;\frac{log_{e}\frac{\alpha }{n_{s}.n_{i}}}{log_{e}\;\left(1-\bar{het}\right)}$$

where n_s_ is the number of SNPs per individual, n_i_ is the number of individuals, α is the percentage of false positive ROH (set to 0.05 in the present study), $\bar{het}$ is the mean SNP heterozygosity across all SNPs. Finally, to ensure low SNP density did not falsify ROH length, the minimum SNP density per ROH was set to 1 SNP every 100 kb and the maximum gap permitted between consecutive homozygous SNPs was set to 250 kb. A minimum ROH length of 1 Mb was set.

Runs of homozygosity were estimated for each individual separately. Each ROH was categorised based on their physical length into 1 to <5 Mb, 5 to <10 Mb, 10 to <15 Mb, 15 to < 20 Mb and ≥20 Mb. For each of the aforementioned ROH length categories, the mean sum of ROH per breed was calculated by summing all ROH per animal in that category and averaging this per breed population. The percentage of SNP residing within an ROH for a given breed, or in the population as a whole, was also calculated by counting the amount of times a SNP appeared in a ROH within the given breed or population whole. To identify the genomic regions most commonly associated with ROH, the top 1% of SNPs observed in an ROH in each breed and across all breeds were selected and adjacent SNPs over this threshold were merged into genomic regions corresponding to ROH hotspots. In addition, the fraction of chromosome residing in ROH was estimated as the mean ROH sum per individual for each chromosome divided by the chromosomal length, as estimated from the SNP coverage.

To determine if the variation in recombination rate across the genome impacted ROH length, ROH were also mapped using the genetic SNP coordinates (i.e. position in the linkage map) available from Johnson et al., [34]. The average recombination rate (cM/Mb) was estimated in 500kb intervals across the genome and also within each ROH hotspot. The percentage of occurrences of a SNP in a ROH was plotted against recombination rate for each chromosome identified as containing a ROH hotspot. In addition, the genetic mapping of ROH length was also used to infer demography using the method proposed by Thompson et al., [35] whereby the map length of a ROH (*l*) = 100/2*g* cM, were *g* is the number of generations of interest. Four ROH length categories were determined so that the analysis would provide information on the effective population size during four different time spans; up to 5 generations ago, 5 to 10 generations ago, 10 to 20 generations ago, and >20 generations ago. The mean sum of ROH per breed was calculated as above and breeds with a larger average abundance of ROH in a particular length class were inferred to have a smaller effective population size during that time span.

### Inbreeding coefficient vs. Runs of homozygosity

The inbreeding coefficient based on ROH (F_ROH_; previously described by McQuillan et al. [4] for cattle), was calculated as the sum of the length of all ROH per animal as a proportion of the total autosomal SNP coverage (2.44 Gb). F_ROH_ was calculated separately as the sum of the lengths of all ROH ≥1 Mb (F_ROH1Mb_), the sum of the lengths of all ROH ≥5 Mb (F_ROH5MB_) and finally as the sum of all ROH ≥10 Mb (F_ROH10Mb_). Pedigree-based inbreeding coefficients (F_PED_) for all animals were calculated using the Meuwissen and Luo [36] algorithm. Depth of pedigree known was measured in complete generation equivalents (CGE) for all animals as described in McParland et al. [37] and Pearson’s correlations between all measures of inbreeding were calculated only for 843 animals with a CGE value ≥6. Each ROH measure of inbreeding was also separately regressed on the pedigree-based inbreeding coefficient for all 843 animals. In addition, two other estimates of inbreeding were calculated (F_GRM_ and F_HOM_) using GCTA [38]. The F_GRM_ was estimated using the VanRaden method [39] based on the variance of the additive genotypes, whereas F_HOM_ was estimated based on the excess of homozygosity following Wright [40].

### Signatures of selection

#### Fst

Global F_ST_ was calculated per SNP using the HierFstat R package [41] with the unbiased estimator proposed by Weir and Cockerham [42] across all breeds. In addition, pairwise F_ST_ was calculated for each pair-wise breed combination (i.e. 6 breeds = 15 comparisons). To reduce noise and identify regions of strong signatures of selection, a sliding window of five SNPs was used to compute an average F_ST_ value of the middle SNP; only the average F_ST_ value is discussed hereon in. Over 90% of all SNPs in a window were within 300 kb of each other and the average length of a window was 231 kb. The empirical P-value for each SNP was then estimated and only the top 0.1% of F_ST_ values (n = 42) were considered to represent a selection signature. To define the boundaries of the identified genomic regions under selection, neighbouring SNPs of the top 0.1% F_ST_ SNPs were included in the selection signature until two consecutive SNPs ranked outside of the top 5% of F_ST_ values [1]. The second SNP that ranked outside of the top 5% of F_ST_ values was not considered in the reported selection signature.

#### HapFLK

To account for the haplotype structure of the populations, as well as varying population effective sizes, the hapFLK statistic [18] was also used to identify possible regions under selection in all breeds. This required the estimation of a neighbour joining tree and a kinship matrix based on a matrix of Reynold’s genetic distances between breeds [19]. The kinship matrix captured the population structure, which was used to model the covariance matrix of the population allele frequencies whereas a multi-point linkage disequilibrium model was used to create local haplotype clusters on each chromosome [43]. The number of haplotype clusters per chromosome was set to 50, which was determined using cross-validation based estimation in fastPHASE [43]. The hapFLK statistic was calculated as the average of 20 expected maximisation iterations. Once hapFLK values were generated for each SNP, P-values were computed based on a chi-square distribution with the python script provided in the hapFLK webpage (https://forge-dga.jouy.inra.fr/projects/hapflk). To limit the number of false positives, a q-value threshold of 0.01 was applied. Local population trees were then re-estimated using only SNPs mapping to the putative selective sweep to identify the population under selection.

### Bioinformatic analyses

Gene annotation of identified selection signatures was completed using Ensembl (http://ensemble.org) and NCBI map viewer (http://www.ncbi.nlm.nih.gov/mapview) on the sheep genome assembly 3.1. Gene ontology (GO) terms that were significantly overrepresented were identified using the software Gorilla (http://cbl-gorilla.cs.technion.ac.il/). Previously reported sheep QTL were obtained from the Sheep QTLdb (http://www.animalgenome.org/cgi-bin/QTLdb/index).

## Results

### Population structure

**Fig 1:**
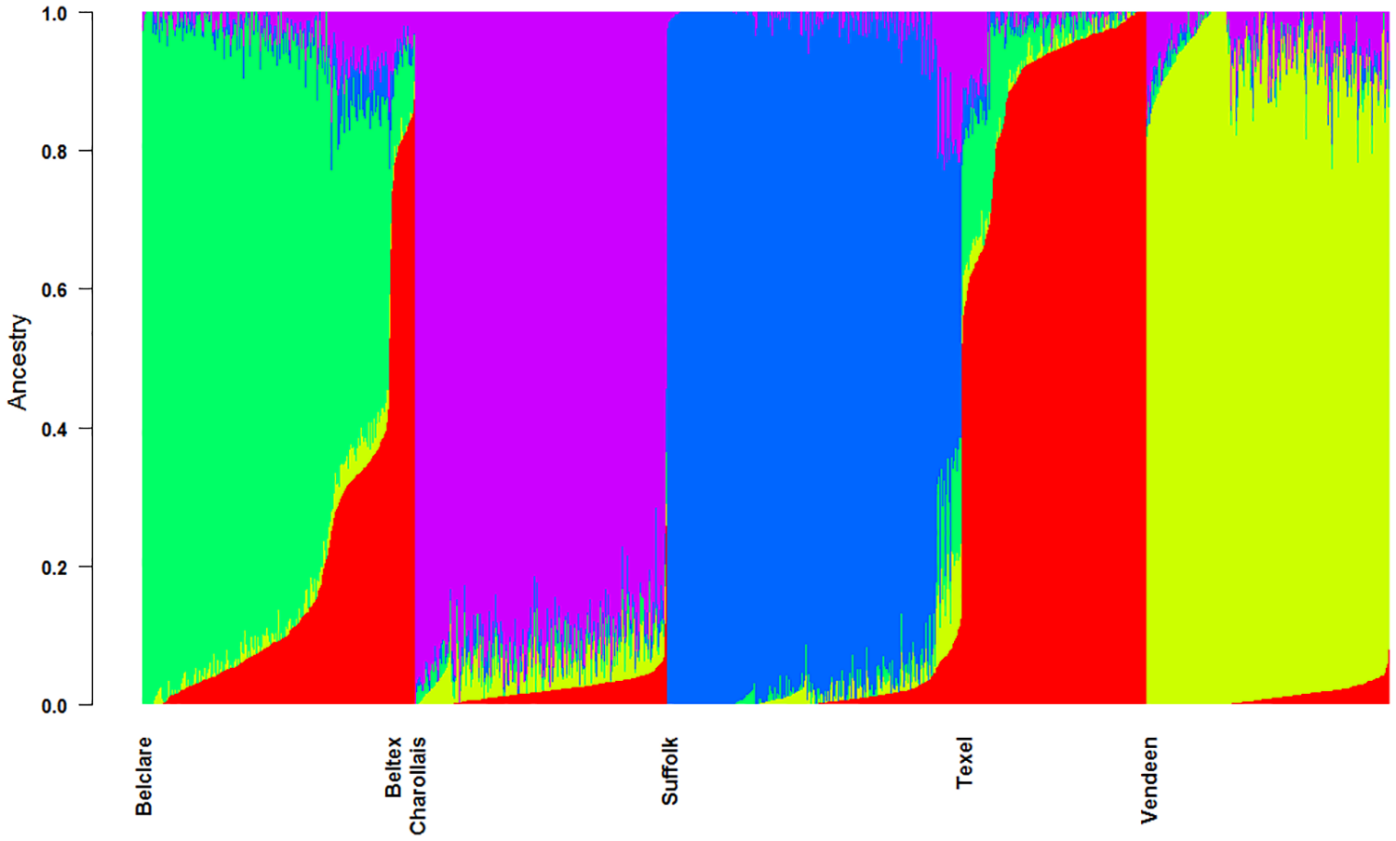
Admixture analysis of six commercial sheep breeds. The number of clusters was set to k = 5.

The principal component analysis in the present study was successful in separating out breed clusters based on genotypic data. The first and second principal components (PC) accounted 37.49% and 32.15% of the variation, respectively (S1 Fig). The Belclare, Beltex and Texel breeds had overlapping clusters, whereas the Charollais, Suffolk, and Vendeen formed distinct separate clusters (S1 Fig). The largest PC separated the Suffolk from the remaining European breeds, whereas the second PC separated the French Charollais and Vendeen breeds into distinct but adjacent clusters. The formation of two clear, non-overlapping clusters for the Suffolk breed is an artefact of the importation of New Zealand Suffolk into the Irish population in recent years. The smaller of the Suffolk clusters represents Suffolk of New Zealand ancestry. The cross-validation error estimates from ADMIXTURE plateaued at K = 5 (S2 Fig), therefore K = 5 was taken as the most probable number of inferred populations. The Belclare was the most admixed of all populations whilst the Suffolk was the least (Fig 1). Evidence of Texel admixture was found in both the Belclare and Beltex populations, with greater evidence detected in the latter.

### Effective population size

**Fig 2:**
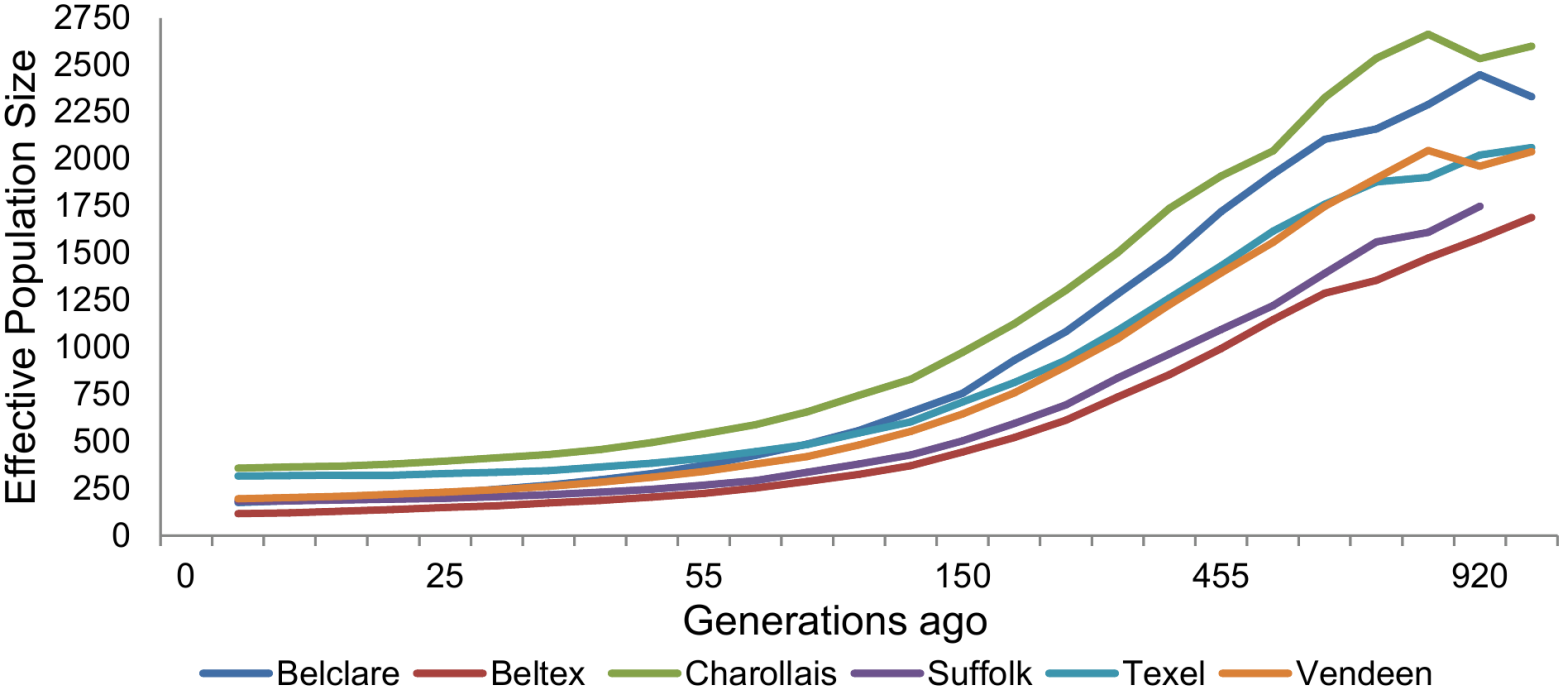
Estimated effective population size across generations for each breed.

The effective population size of all six breeds declined over time (Fig 2). Based on the sample population used in the present study, the Charollais breed had the largest effective population size across all generations, whereas the Beltex had the smallest. Assuming a generation interval of four years, the estimated effective population size in the last 50 years ranged from 115 in the Beltex breed to as high as 357 in the Charollais breed.

### Runs of homozygosity

**Fig 3:**
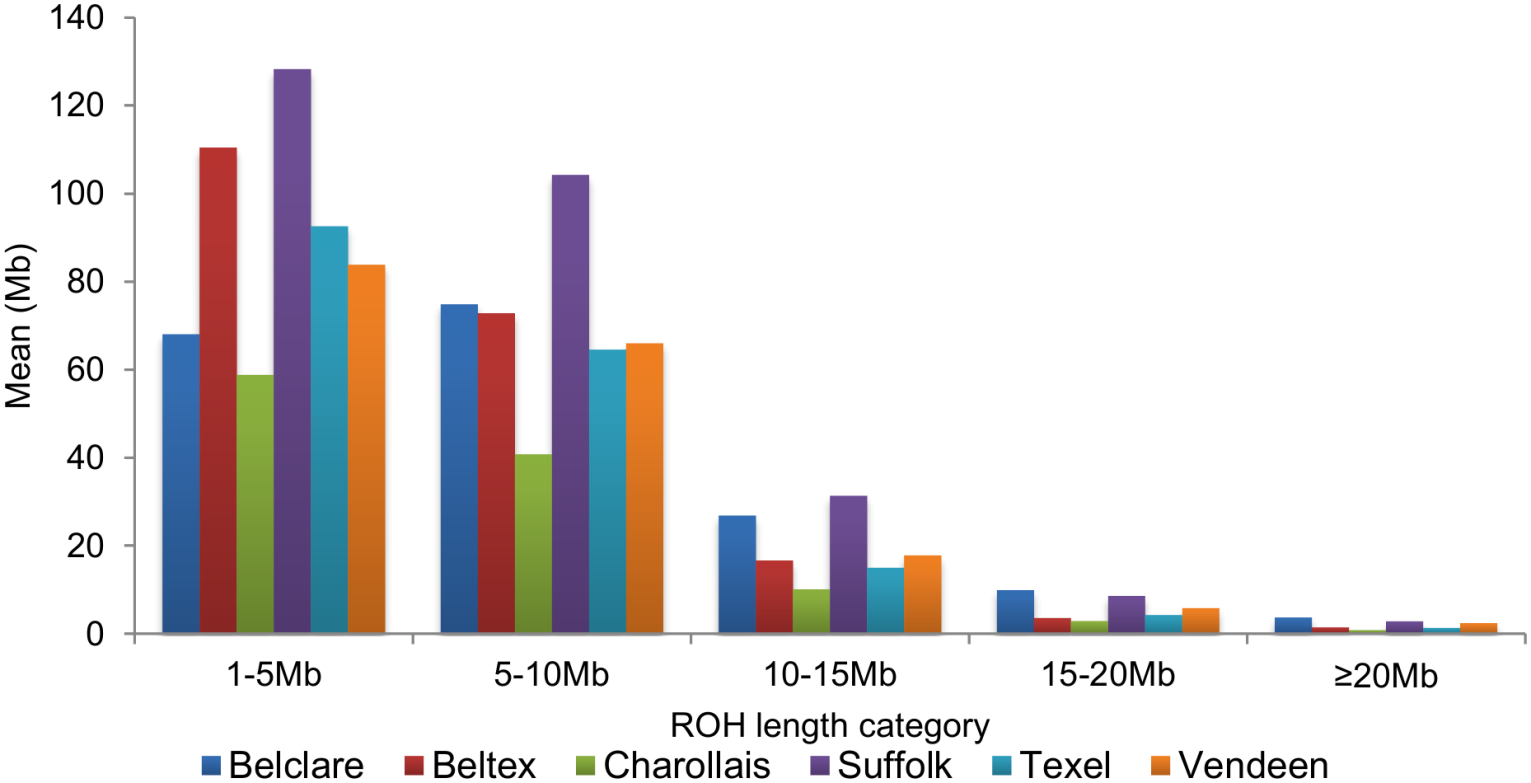
The mean sum of runs of homozygosity (ROH) per animal within each ROH length category.

ROH were common across all breeds, although the length and frequency of ROH often differed per breed. ROH were identified in all animals with the exception of one Vendeen animal. The Suffolk and Beltex breeds had a greater mean proportion of their autosome, 0.053 (128.31 Mb) and 0.045 (110.47 Mb), respectively, covered in shorter ROH (1 - <5 Mb) in comparison to the other four breeds; mean ROH autosomal coverage per breed ranged from 39.94 to 92.61 Mb in the remaining breeds (Fig 3). For all breeds, the majority of detected ROH were less than 10 Mb in length, with relatively few long ROH ≥20 Mb detected within each breed (mean ROH coverage per breed for ROH ≥20 Mb ranged from 0.83 to 3.7 Mb). In fact, only 8.21% of the individuals had at least one ROH ≥20 Mb in length and these were primarily in the Belclare breed; 13.99% of the Belclare individuals had at least one ROH ≥20 Mb in length.

ROH were also mapped using their genetic positions and the abundance of ROH in different length classes was used to qualitatively evaluate the historical demography of each of the breeds. The time to the most recent common ancestor (TMRCA) was estimated for four different categories for each breed in S3 Fig. The ability to infer demography for > 20 generations ago was limited by the density of the SNP panel. ROH were more abundant in all TMRCA categories in the Suffolk population. This suggests that the effective population size in the Suffolk was small both in recent and past generations. The substantial increase in the abundance of ROH in the Belclare breed from 10 to 20 generations ago to <5 generations ago, suggests a recent decrease in the effective population size. The lower ROH abundances found in the Charollais population suggests a relatively large effective population size has been maintained across generations.

**Fig 4:**
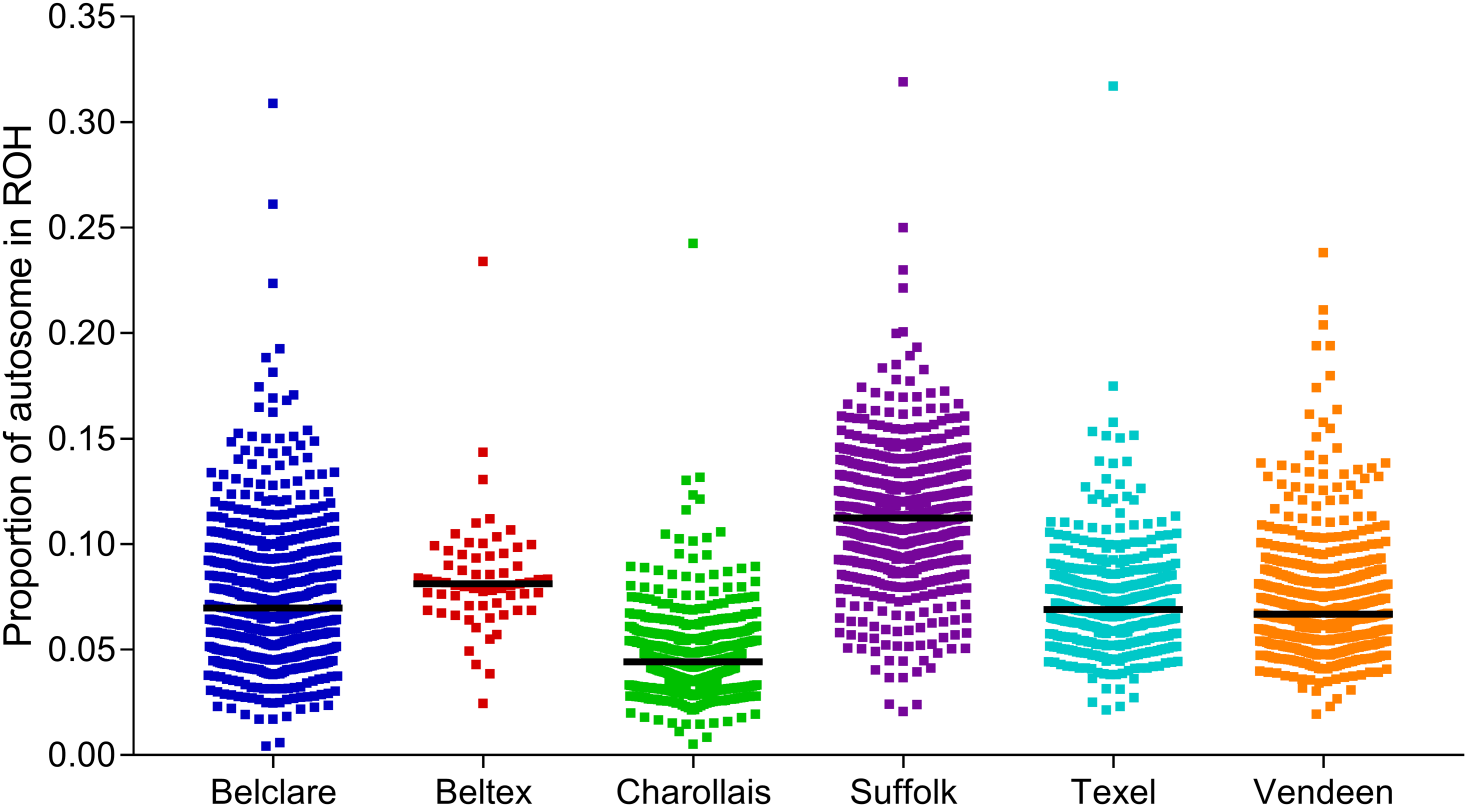
Proportion of autosome covered in runs of homozygosity (ROH) per animal. The black line indicates the median ROH sum per individual within each breed.

The proportion of the autosome covered in ROH varied both within and across breeds (Fig 4). The Charollais breed had a tendency towards fewer ROH, whereas large inter-animal variability existed within the Suffolk breed; individual ROH autosome coverage ranged from 0.025 (50.53 Mb) to 0.319 (778.69 Mb) within the Suffolk population. The three most homozygous animals in the sample population used in the present study had, on average, 0.315 (768.65 Mb) of their autosome covered in ROH, equivalent to almost a third of their genome (Fig 4).

**Fig 5:**
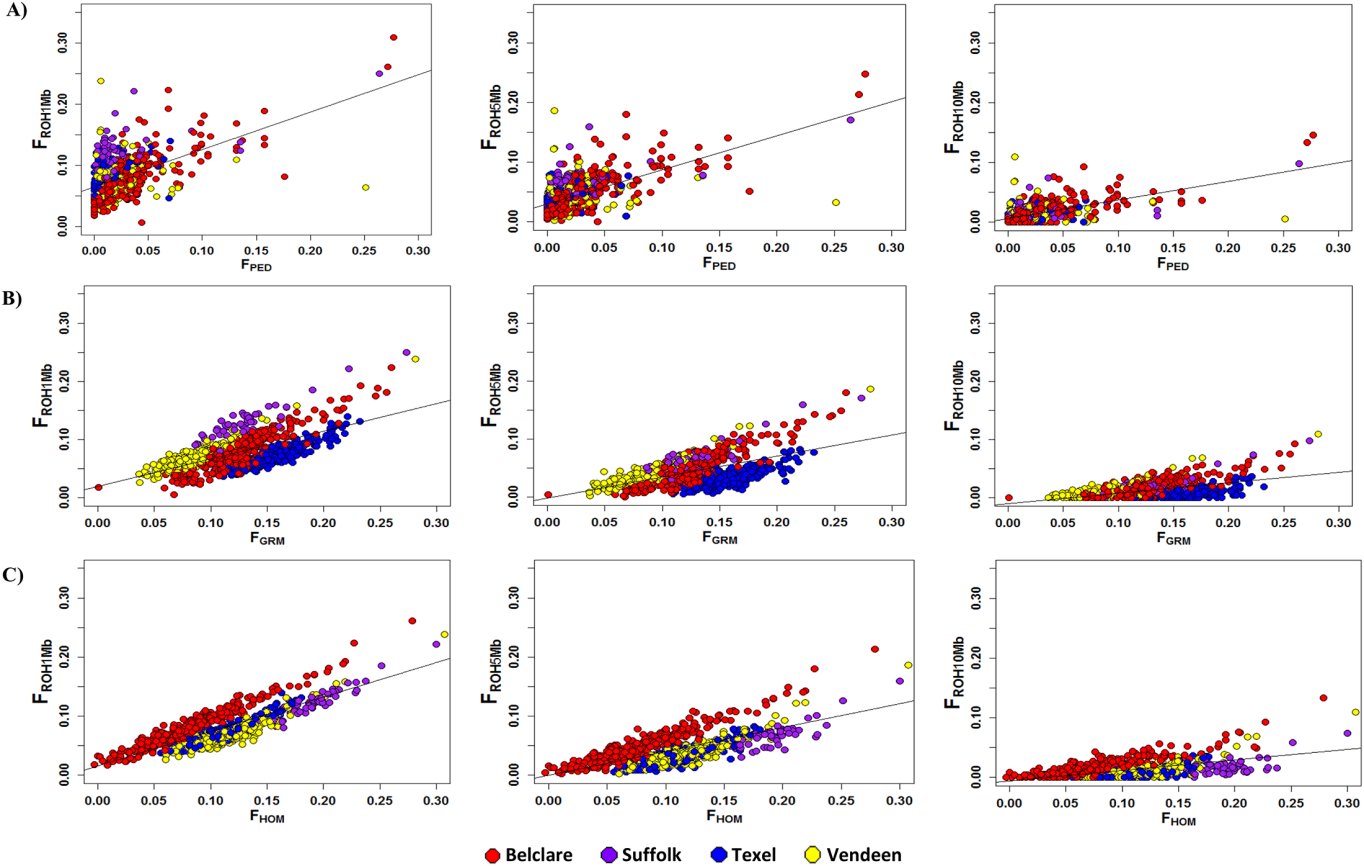
Matrix of scatter plots for different measures of inbreeding calculated from runs of homozygosity (F_ROH_) versus inbreeding estimated from A) pedigree analysis (F_PED_), B) genomic relationship matrix (F_GRM_) and C) the observed versus expected homozygotes (F_HOM_) on 843 animals with at least 6 complete generation equivalents. Four populations were included in the analysis; Belclare (red), Suffolk (purple), Texel (blue) and Vendeen (yellow).

Moderate to weak correlations per breed existed between the pedigree inbreeding coefficient and the varying ROH inbreeding measures (Table 1). The lowest correlations between F_PED_ and F_ROH_ were found in the Vendeen population whilst the strongest existed in the Belclare population. The Pearson correlations between F_PED_ and F_GRM_ were low in the Vendeen population (0.18, P-value <0.01) and moderate in the Belclare (0.70, P-value <0.001), Suffolk (0.51, P-value <0.001) and Texel (0.51, P-value <0.001) populations. Similar correlations existed between F_PED_ and F_HOM_ (Beclare 0.73, P-value <0.001; Suffolk 0.54, P-value <0.001; Texel 0.49, P-value <0.001; Vendeen 0.16, P-value <0.05). The correlations between F_GRM_ and F_ROH,_ and F_HOM_ and F_ROH_ were higher than those between F_ROH_ and F_PED._ The intercept of the regression of all ROH inbreeding measures on F_PED_ was greater than zero, suggesting that the F_PED_ may underestimate genome homozygosity (Fig 5). The smaller intercept of F_ROH10Mb_ is consistent with longer ROH arising from more recent inbreeding that is more likely to be captured by pedigree recording.

**Table 1:** The correlation between runs of homozygosity (ROH) based inbreeding coefficients and inbreeding coefficients estimated from pedigree (F_PED_), the genomic relationship matrix (F_GRM_) and the observed versus expected homozygotes (F_HOM_). Three different ROH inbreeding measures were used which corresponded **to** the minimum length of the ROH used in the estimation (F_ROH1Mb_, F_ROH5Mb_, F_ROH10Mb_).

	Belclare	Suffolk	Texel	Vendeen
Number of animals	304	53	248	238
r(F_PED-_ F_ROH_)				
>F_ROH1Mb_	0.76***	0.54***	0.52***	0.15*
>F_ROH5Mb_	0.75***	0.55***	0.47***	0.15*
>F_ROH10Mb_	0.71***	0.58***	0.41***	0.12
r(F_GRM_-F_ROH_)				
>F_ROH1Mb_	0.92***	0.93***	0.90***	0.94***
>F_ROH5Mb_	0.91***	0.88***	0.79***	0.91***
>F_ROH10Mb_	0.84***	0.86***	0.60***	0.83***
r(F_HOM_-F_ROH_)				
>F_ROH1Mb_	0.98***	0.97***	0.91***	0.93***
>F_ROH5Mb_	0.96***	0.88***	0.80***	0.89***
>F_ROH10Mb_	0.87***	0.79***	0.62***	0.80***

* P-values <0.05;

** P-values <0.01;

*** P-values <0.001

**Fig 6:**
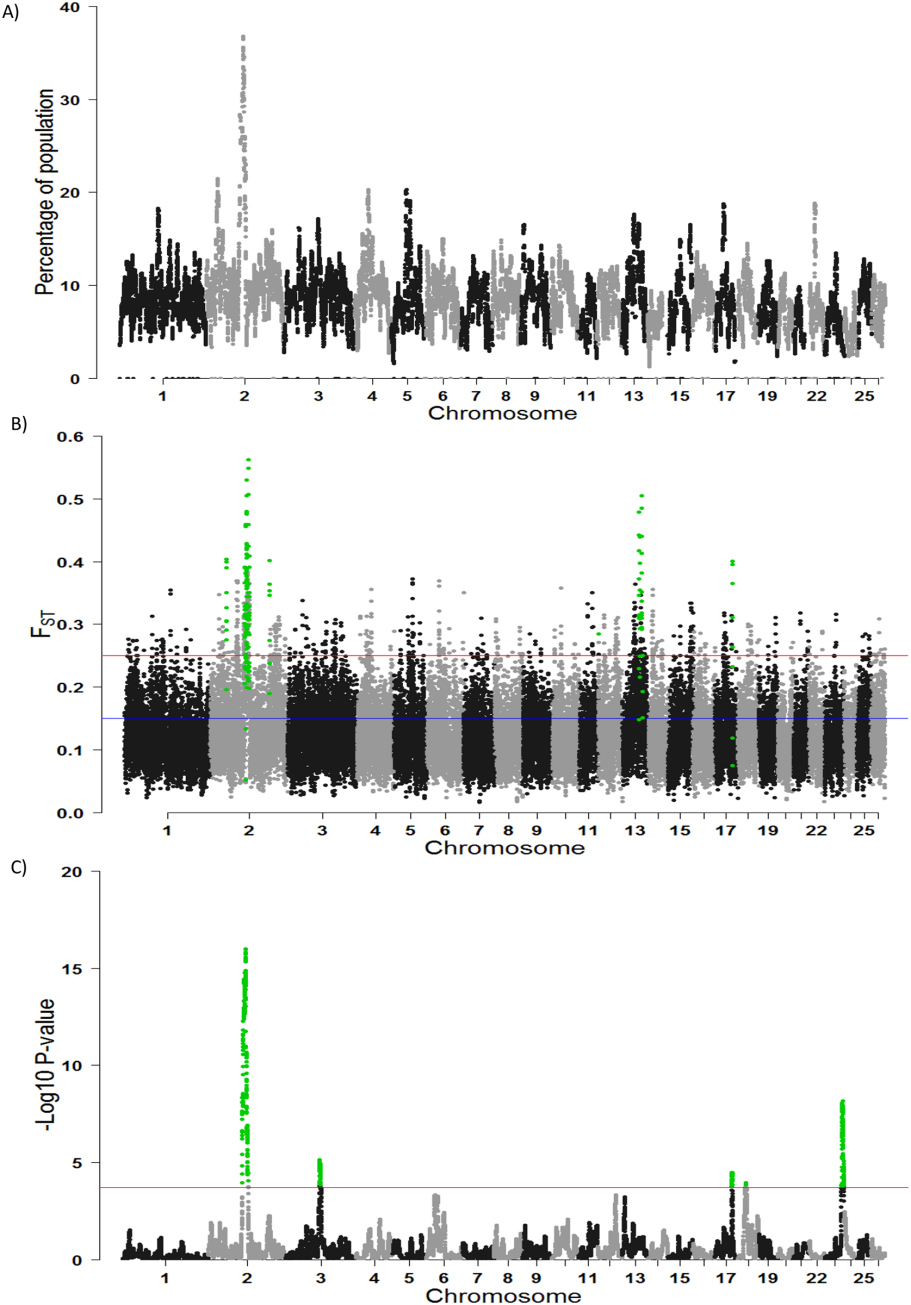
Genomic regions detected to be under divergent selection across all breeds. A) The frequency of a single nucleotide polymorphism (SNP) in a run of homozygosity (ROH) B) global F_ST_ values across all breeds where the blue line indicates SNPs that exhibited great differentiation and the red line indicates SNPs that exhibited very great differentiation C) a haplotype-based hapFLK test where the red line indicates the significance level threshold of 0.0001. SNPs highlighted in green are those identified within putative selection signatures.

The percentage of the autosome residing in a ROH varied by chromosome and by breed, ranging from as low as 1.64% of OAR24 in the Charollais population to as high as 14.21% of OAR15 in the Suffolk population (S4 Fig). Several genomic regions were identified that frequently appeared in a ROH within individual animals (Fig 6A), although the region harbouring ROH often differed per breed (S5 Fig). The top 1% of SNPs with the highest occurrences in a ROH across and within all breeds, were identified as candidate SNPs under directional selection. All adjacent SNPs over this threshold were merged to form ROH islands and in total, 11 genomic regions under putative directional selection across all breeds were identified on OAR 2, 4, 5, 17 and 22 (Table 2). The ROH hotspot with the highest occurrences was located on OAR2 (115.48–126.34 Mb) and likely candidate genes within this region include *MSTN*, *ITGAV*, *BIN1* and *NUP35*, all of which are involved in muscle differentiation. Within breed, this region on OAR2 (115.48–126.34 Mb) was identified as under putative selection in the Belclare, Beltex and Texel populations (S1 Table). In the Charollais population, several regions under putative selection were identified on OAR 2, 4, 9 and 23, and plausible candidate genes within these regions included the fertility related genes *NTRK2*, *HECW2*, *STK17B* and *ITGB8*. Although more regions were identified as under putative selection in the Vendeen population, the occurrence of a SNP in a ROH was much lower in the Vendeen population in comparison to the other populations (S5 Fig), with the strongest signal on OAR2 only detected in 37.52% of individuals. These ROH hotspots within and across breeds were found to frequently coincide with regions of very low recombination rate (Table 2; S6 Fig; S1 Table). To test if these ROH hotspots were likely a result of the combination of selection and inbreeding, the occurrence of a SNP in a ROH was correlated with the SNP global F_ST_ value and–log_10_ hapFLK p-value (S7 Fig). Significant moderate correlations were found between each selection signature method and the occurrence of SNP in a ROH (F_ST_-SNP in a ROH 0.25, <0.0001;–log_10_ hapFLK p-value-SNP in a ROH 0.37, <0.0001).

**Table 2:** Runs of homozygosity (ROH) hotspots across all breeds, as defined as the top 1% of SNPs that occurred in a ROH. The number of SNPs within these hotspots are listed, as well as the average recombination rate (cM/Mb) within each hotspot and the putative candidate genes under selection.

OAR	Position (Mb)	No Sig SNP	Number of genes	Hotspot cM/Mb	Candidate genes	Gene function
2	35.78–35.93	4	3	0.00	UBQLN1	Immunity
2	36.08–39.59	53	60	0.82	EBF2	Adipocyte development
2	107.77–111.34	69	16	0.31	-	-
2	115.48–122.52	135	60	0.49	MSTN	Muscle differentiation
2	123.36–126.34	65	12	0.18		
4	44.48–47.29	48	30	0.50	RELN	Nematode resistance
5	47.02–48.82	27	36	0.14	EGR1	Fertility
5	49.38–49.90	9	15	0.52	-	-
5	59.73–60.00	5	5	0.00	GPX3	Nematode resistance
17	29.07–29.14	3	0	0.00	-	-
22	19.39–19.50	2	1	0.00	HPSE2	

### Selection signatures

#### F_ST_

Several genomics regions with high F_ST_ values were detected across all breeds (Fig 6B). The mean genomic F_ST_ value across all SNPs was 0.127, indicating moderate genetic differentiation according to Wright’s classification. In total, 11 different selection signatures were identified on OAR2, OAR13 and OAR17. The highest ranked SNPs were located on OAR2 in two genomic regions between 121,455 and 123,362 kb and between 115,476 and 117,369 kb (Fig 5B); several genes were identified within each of these regions on OAR2 (Table 1) including *ITGAV* and *BIN1*, respectively. Of the 42 significant F_ST_ values, 24 of these were located directly within genes and a total of 144 genes were identified within the selection signatures boundaries (Table 3). Gene ontology (GO) terms associated with the 144 genes were tested for evidence of functional enrichment. This revealed enrichment for GO terms associated with negative regulation of the JNK cascade (GO:0446329; P-value = 7.57x10^-4^) and the opioid receptor signalling pathway (GO:0038003; P-value = 9.13x10^-4^). Likely candidate genes identified with the putative selection signatures included those involved in skin pigmentation and coat colour (*ASIP*, *EDN3*, *HERC2*), body size and muscle formation (*KSR2*, *NUP35*, *BIN1*, *ITGAV)*. In addition, non-coding DNA sequences and multiple uncharacterised genes were identified within these regions of selection, including LOC101106402 and LOC101115300 which may be the putative genes under selection within the selective sweeps on OAR2 (113,014 and 114,763 kb) and OAR13 (52,652 and 53, 111 kb). Orthologues of LOC101106402 and LOC101115300 include the human *ARPC4* (ENSG00000241553) and bovine *SIRPB1* (ENSBTAG00000039520), respectively.

**Table 3:** Detected selection signatures containing the top 0.1% of SNP, ranked on F_ST_. Detailed are the number of significant (Sig) SNP (P-value <0.0001) within each selection signature, the maximum (Max) F_ST_ value of a SNP within this signature, the number of genes identified within the selection signature boundaries and the identified candidate gene and its function.

OAR	Position (Mb)	Number Sig SNP	Max F_ST_	Max F_ST_ SNP	Number Genes	Previously reported	Candidate Genes	Gene Function
2	121.46–123.26	5	0.56	rs405734194	8	[20]	ITGAV	Adipogenic differentiation [44]
2	115.48–117.37	16	0.53	rs416646426	21	[20]	BIN1	Muscular differentiation [45]
13	61.59–63.45	4	0.51	rs411530656	37	[1]	ASIP	Pigmentation [46]
13	52.65–53.11	3	0.48	ss836362476	9	-	LOC101115300	Pigmentation; Melanocortin receptor 3-like
2	113.01–114.76	2	0.46	rs405194800	10	[20]	LOC101106402	Muscle; Actin related protein
13	56.22–56.59	2	0.44	rs410610128	7	[20]	EDN3	Pigmentation [47]
2	125.16–125.52	2	0.41	rs424708539	4	-	NUP35	Muscular differentiation [48]
2	52.03–52.64	3	0.40	rs409342111	28	[1, 20, 21]	NPR2	Skeletal morphology & body size [49]
2	191.31–191.58	1	0.40	rs426713055	2	-	GYPC	Immunity [50]
17	56.43–56.86	2	0.40	rs398360598	6	-	KSR2	Body weight [51]
2	110.97–112.55	1	0.39	rs401443461	13	-	OCA2,HERC2	Pigmentation [52]

Pairwise breed F_ST_ analyses also identified several genomic regions that were highly differentiated between pairs of breeds. The Beltex and Suffolk breeds had the largest number of putative selective sweeps (18) between all breed comparisons and a greater mean genomic F_ST_ value across all SNPs (0.087) than all other pairwise breed comparisons (S8 and S9 Figs). The two strongest differentiated regions between the Beltex and Suffolk breed were located on OAR25 (34,508–34,845 kb; maximum F_ST_ SNP = 0.71) and OAR14 (16,062–16,464 kb; maximum F_ST_ SNP = 0.69), overlapping the zinc finger *ZMIZ1* on OAR25 and the ATP binding cassettes *ABCC12* and *ABCC11*. The same genomic region on OAR2 between 121,776–123,077 kb surrounding *FSIP2* and *TMED2*, was highly differentiated between the Texel vs. the Charollais, Suffolk and Vendeen breeds. The least differentiated breed comparison was the Beltex vs. Texel, where the mean genomic F_ST_ value across all SNPs was 0.025 (S8 Fig). The strongest differentiated region between the Beltex and Suffolk breed was located on OAR2 (205,132–205,403 kb) surrounding the novel gene ENSOARG00000018335.

#### hapFLK

**Fig 7:**
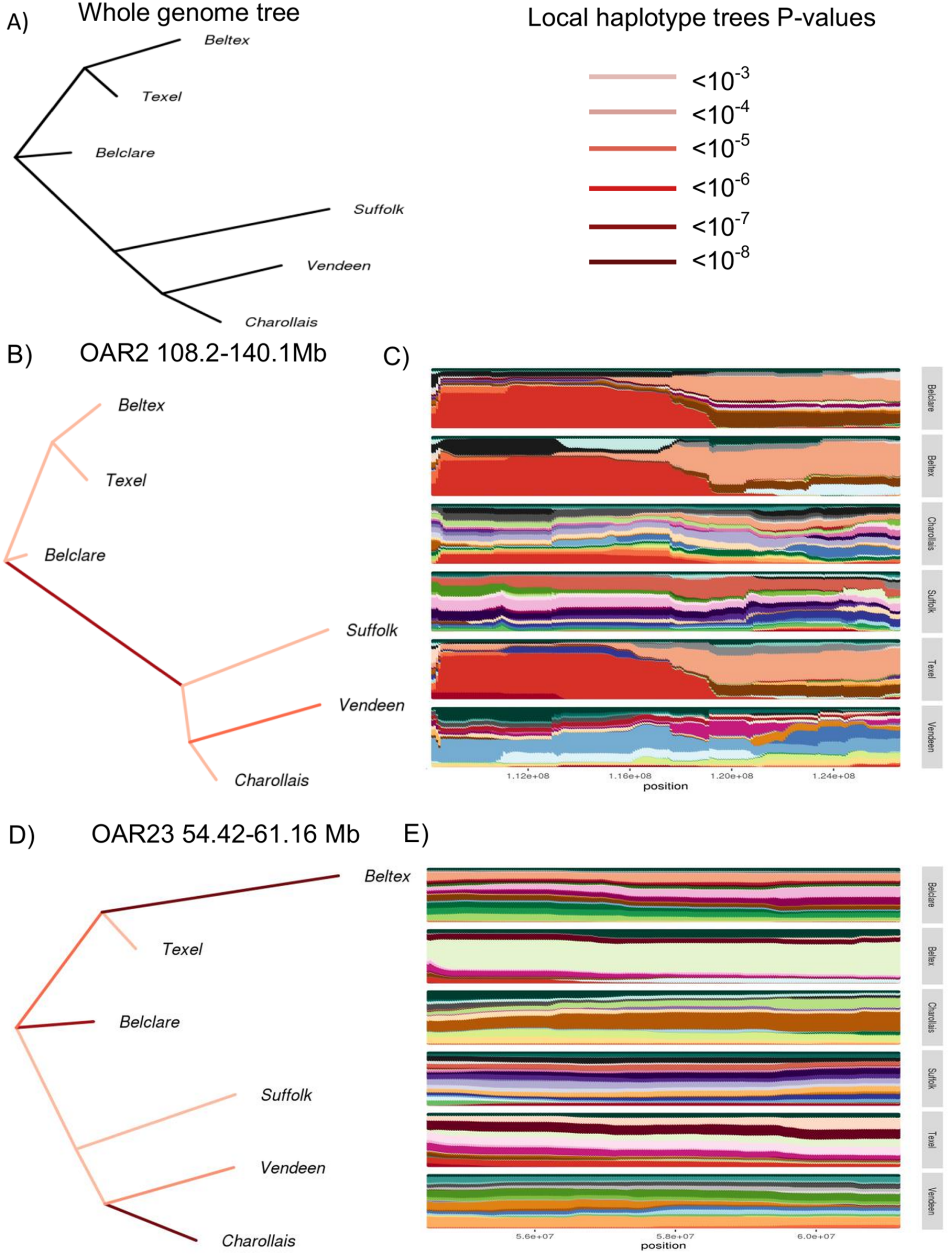
Local population tree estimated in two selection signatures identified on OAR2 and OAR23. A) The whole genome population tree B & D) The local population tree re-estimated using only single nucleotide polymorphisms (SNPs) identified within the putative selection signature C & E) The haplotype clusters for the selection signature.

Five significant regions (P-value <0.0001) were detected as under selection signatures on OAR2 (108,265–126,623 kb), OAR3 (108,302–111,442 kb), OAR17 (54,392–55,644 kb), OAR18 (27,798–28, 167 kb) and OAR23 (54,419–61,155 kb) (Fig 6C). Of these five selection signatures, only the region on OAR2 was consistent with the F_ST_ analysis. Multiple genes were identified within each of these five selection signatures (Table 4) including *MSTN* on OAR2 and *MC4R* on OAR23, both of which enhance growth performance. The breed(s) under selection for each of the four selection signatures were identified through comparison of the local population trees to those estimated from whole genome data (Fig 7). The Suffolk, Vendeen and Charollais breeds were significantly differentiated from the Belclare, Beltex and Texel breeds for the selection signature identified on OAR2, presumably due to the selection for mutations within the myostatin gene that contribute to muscle hypertrophy within the latter breeds. The SNPs within this region on OAR2 were almost fixed in the Belclare, Beltex and Texel populations in the present study, which could be indicative of a hard sweep signal. In contrast, no evidence for a hard selective sweep was found for the selection signature on OAR23 (54,419–61,155 kb), suggesting this genomic region has been subjected to a recent selection pressure. The reduced, albeit different, haplotype diversity for this selection signature on OAR23 (Fig 7) in both the Beltex and Charollais populations, suggests that selection for this genomic region started on different haplotype backgrounds. Selection for this genomic region on OAR23 may be for variants within the melanocortin-4 receptor (*MC4R*) which has been associated with body weight in sheep.

**Table 4:** Putative selective sweeps identified in the hapFLK-based analysis.

OAR	Position (Mb)	Number of SNP	Peak P-value	Peak Q- value	Number of genes	Previously reported	Candidate gene	QTL
2	108.24–126.62	303	1.02x10^-16^	1.19 x10^-12^	119	[18, 20]	MSTN	Carcass weight and conformation [53]
23	54.02–61.56	119	6.97x10^-9^	1.30 x10^-6^	109	[20]	MC4R	Growth and meat quality traits [54]
3	108.19–111.74	77	7.42x10^-6^	7.03 x10^-4^	68	-	TRHDE	Growth traits [55]
17	53.94–55.68	29	3.22x10^-5^	2.58 x10^-3^	21	-	SH2B2	Growth traits [56]
18	27.79–28.17	9	1.11 x10^-4^	7.75 x10^-3^	3	-	PCSK6	Fertility [57]

## Discussion

The identification of genomic regions that are under the influence of both natural and artificial selection can help determine the genetic basis of economically important traits that are segregating within or between breeds. Several genomic regions under putative selection were identified in the present study across six commercial sheep breeds and these signatures provide an insight into the genes contributing to their diverse phenotypes. However, it is important to acknowledge that regions identified as putative selective sweeps should be interpreted cautiously as differences in demographic history such as genetic drift, effective population size, inbreeding and population bottlenecks can also result in false positive signatures of selection [23].

### Demographic history

The observed decline in the effective population size over time in the breeds considered in the present study coincides with human-mediated specialisation for wool and milk traits 4,000–5,000 years ago [58]. The reduction in the effective population size to hundreds in recent years is consistent with population subdivision and selection, but also the relatively limited use of artificial insemination [1]. However, unlike cattle [37], sheep have retained a relatively high level of genetic diversity. In general, our estimates of effective population size in the present study are similar to those previously reported in comparable breeds of sheep [1–3]. The smallest effective population size in the Beltex breed may reflect the small founding population. The Beltex breed, which was developed in Belgium by selectively breeding Texel sheep for double-muscling, was only introduced into the United Kingdom in 1989, where they acquired their name and have since been refined to the modern form. The genetic similarity between the Beltex and Texel populations is evident from the ADMIXTURE and PCA results (Fig 1 and S1 Fig). The high abundance of ROH in the Beltex breed with a TMRCA between 5 to 10 and 10 to 20 generations is consistent with that of a small effective population size during breed formation [59], and suggest the presence of ancient relatedness possibly occurring from a population bottleneck. Similarly, the large abundance of ROH across all TMRCA categories in the Suffolk population is consistent with the low effective population size we detected in this breed. Kijas et al., [1] previously reported a mean genomic inbreeding coefficient of 0.22 in the Irish Suffolk breed suggesting a high level of relatedness among this breed. The consistent overlap between the estimated effective population size and the abundance of ROH within each TMRCA category across all breeds suggests that ROH can be successfully applied to infer demography within sheep populations.

### ROH as a predictor of ovine inbreeding

In the absence of pedigree information, previous studies have documented the usefulness of the sum of an individual’s ROH coverage to infer the inbreeding level of an individual [5, 7, 9, 59]. The moderate correlation in the present study between F_PED_ and F_ROH_, with the exception of the Vendeen population_,_ further substantiates the usefulness of ROH as a measure of inbreeding in a population. The moderate correlations in the present study may be partly explained by the relatively shallow depth of the pedigree records for all breeds (Mean CGE = 6.5). Similar correlations between F_PED_ and F_ROH_ were previously reported in various cattle populations [5–7, 60]. However, it is important to acknowledge that F_PED_ is often an imprecise measure of the proportion of the genome that is identical-by-descent (IBD) as it is limited by pedigree depth, pedigree errors and linkage [61], and fails to account for the variability that can exist in IBD estimates between individuals of the same pedigree [62]. Therefore two further estimates of genomic inbreeding, F_GRM_ and F_HOM,_ were used to evaluate the efficacy of F_ROH_ as a measure of inbreeding in sheep populations. The strong correlation between F_ROH1Mb_ and F_HOM_ in the present study corroborates previous results found in cattle [7, 63], although the correlation between F_ROH_ and F_GRM_ was greater than those previously estimated [7, 60, 63]. Both measures, F_GRM_ and F_HOM,_ have been previously shown to be strongly dependent on allele frequencies, particularly for populations with divergent allele frequencies, which can lead to misleading IBD results [7]. The moderate to strong correlations between F_ROH_ and all inbreeding measures in the present study suggests the extent of a genome under ROH can be used to accurately predict the proportion of the genome that is IBD in sheep populations. Nevertheless, it must be acknowledged that these correlations are likely to underestimate the precision of each of these inbreeding estimators due to their joint correlation with IBD (e.g. r(F_ROH_, F_GRM_) ≈ r(F_ROH_, F_IBD_)*r(F_GRM_,F_IBD_)). In addition, it should be underlined that not every ROH is attributable to IBD and instead possibly originated from identity-by-state due to localised low levels of recombination and high levels of linkage disequilibrium in unrelated ancestors [4].

### Genomic regions of selection

The geographic adaptation and selection for specialised production traits has resulted in many shared but also breed-specific phenotypes in sheep. Here we used two different, yet complementary, statistical approaches (i.e., F_ST_ and HapFLK) to identify putative selection signatures across six phenotypically different commercial breeds. The hapFLK analysis identified fewer selection signatures in the present study than F_ST_, but five out of the seven selection signatures identified on OAR2 using F_ST_, were located within the 18.36 Mb region identified as under selection using hapFLK. In addition, hapFLK is also expected to be more stringent than F_ST_, which typically suffers from bias and false positives; this is because hapFLK accounts for the haplotype and structure of the population [18]. To limit the number of false positives identified using F_ST_ in the present study, only the top 0.1% of F_ST_ values were considered as representing a signature of selection which is consistent with studies undertaken elsewhere [1, 16, 17].

Global F_ST_ was used in the current study to identify selection signatures that were differentially fixed across breeds and to determine how selection altered the allele frequency patterns between these breeds. The content of these differentiated regions strongly suggest selection for genes that are associated with skin pigmentation, body size and muscle formation. Skin pigmentation type has been a selection criterion of sheep breeders since ancient times [64]. Candidate genes identified in selection signatures in the present study that are involved in the development and migration of melanocytes in skin pigmentation included *EDN*, *HERC2* and *OCA2* [47, 52]. In addition, *ASIP* whose duplication has also been previously shown to control a series of alleles for black and white coat colour in sheep [46] was also identified. Although none of the breeds used in the present study traditionally have the black coat phenotype, black skin colour is an important characteristic of the Suffolk breed. Indeed the allele frequency between the Suffolk and the other breeds differed substantially within these selected regions. Moreover, positive selection for regions harbouring these genes has been previously reported in sheep by Kijas et al., [1] and Fariello et al., [20].

The objective of selective breeding programs to increase the productivity and profitability of the sheep meat industry, has contributed to the differentiation of several genomic regions that enhance muscling and weight-gain across breeds [65]. Several candidate genes associated with such traits were identified within selection signatures in the present study, including *NPR2* on OAR2 which is involved in skeletal morphology and body size, and has been previously identified by both Kijas et al., [1] and Moradi et al., [21] to reside in a selection signature in sheep. The integrin subunit, *ITGAV*, identified within the selection signature on OAR2 from 121,455 to 123,259 kb, has also been reported to play a key role in adipogenic differentiation of human adipose tissue stem cells [44]. The accumulation of adipose tissue is often a characteristic of increased body weight in sheep. Furthermore, *KSR2*, commonly known as “The fat gene”, was also identified within a selection signature on OAR17. Variants within *KSR2* have been shown to play an important role in energy homeostasis and obesity in humans [51]. The hapFLK analysis also identified a putative selection signature on OAR17 in close proximity to *KSR2* (<800 kb). This region included the *SH2B2* gene which has been previously associated with growth performance in cattle [56]. The identification of two selection signatures by two different methods within close proximity, suggests that the 53.94–56.86 Mb region on OAR17 may be under putative selection for growth related genes.

The intense selection for enhanced muscle development within the Beltex and Texel breeds may be why several of the selection signatures included candidate genes that are essential to muscle differentiation. Two such candidate genes involved in muscle differentiation include *BIN1* and *NUP35*. Variants within *BIN1* and *NUP35* on OAR2 have been previously associated with muscle myopathy in humans and mice, although their role in sheep is unknown [66, 67]. However, it most likely that the nucleoporin *NUP35* plays an important role in myogenic differentiation through the formation of the nuclear pore complex [49]. Similarly, *BIN1* expression, structure and localisation is known to be tightly controlled during muscle differentiation, suggesting *BIN1* is a key regulator in the formation of muscular tissue [45]. Selection surrounding myostatin, the causative gene for the characteristic double muscling of the Texel and Beltex breeds [53], was not detected in the present study to be in a selection signature based on the global F_ST_ values but was significantly differentiated in the pairwise F_ST_ comparison between the Texel population and both the Suffolk and Vendeen populations and between the Belclare and Charollais populations. Similarly, the hapFLK analysis identified a hard selective sweep on OAR2 (108,265–126,623 kb) within the Belclare, Beltex and Texel populations, most likely acting on the myostatin gene *MSTN*. The intense selective breeding for muscle hypertrophy within the Texel breed has resulted in the fixation of multiple alleles within *MSTN* in that breed [53]. The origin of the Beltex from the Texel breed, and indeed the infusion of Texel blood into the Belclare population in recent years, has most likely resulted in the continued selection of the favourable mutations of *MSTN* within these breeds. The overlap of the five global F_ST_ selection signatures and the hapFLK selection signature on OAR2, suggests that this 18.36 Mb genomic region has been selectively targetted for the many genes within this region that are impact sheep growth and muscle formation, and has been previously identified as a selection signature by Fariello et al., [18].

Although plausible candidate genes were identified in several selection signatures in the present study, many non-coding regions and uncharacterised genes also resided within these regions that cannot be dismissed. Further annotation and investigation of the functional properties of these uncharacterised genes is necessary, as they may contribute to phenotypic variability in performance traits, or traits associated with disease resistance or environmental adaptations. The identification of two selection signatures on OAR2 and OAR13 in the present study were the most likely candidate gene is uncharacterised, further substantiates the need for more comprehensive annotation of the sheep genome. Animal QTLdb identified several QTL for carcass muscle weight, parasite resistance and meat quality that overlapped these signatures of selection [68–70].

Overlapping ROH are often identified across individuals due to the selection of common ancestors that carried superior alleles at specific locations [9]. This is evident in the Belclare, Beltex and Texel populations in the present study, where more than 50% of the individuals within each of these populations contain a ROH overlapping *MSTN*. The identification of regional selection for adaptive variants using the distribution of ROH has been successfully applied elsewhere [8, 9, 71]. Indeed, Kim et al., [9] identified that two-thirds of the selection signatures identified in a German Holstein population overlapped with high ROH regions in U.S. Holsteins. In the present study, 9 of the 11 selection signatures identified using global F_ST_ contained SNPs that appeared in a ROH in more than 15% of the animals in the study (Fig 5A). However, when focusing on the top 1% of SNPs with the highest occurrence in a ROH as ROH hotspots across all breeds, only two of the ROH hotspots identified (both on OAR2) overlapped with those identified using global F_ST_ and one with the hapFLK method. Previous work by Pemberton et al., [72], and Bosse et al., [73] have demonstrated that ROH distributions are not uniform and instead have distinctive continental patterns. The existence of ROH hotspots and coldspots therefore has been partly attributed to the variation in recombination events and GC content across the genome and not solely selection. A similar trend was detected in the present study whereby ROH hotspots frequently coincided with regions of low recombination rate. This high ROH abundance in low recombination regions may have been partially attributed to by selection, however decreased SNP density and increased nucleotide diversity in regions with high recombination may have attributed to this abundance [74]. Despite this, the significant correlation between the occurrence of ROH in a SNP (S7 Fig) and the global F_ST_ per SNP in the present study and elsewhere [75], supports the hypothesis that the observed ROH patterns are not solely the result of demography and instead harbour targets of positive selection. Therefore it may be possible to use the distribution of ROH across the autosome to limit the number of false positives identified using the global F_ST_ method.

## Conclusion

In conclusion, changes in autosome autozygosity, allele frequency patterns, and the extent of recombination across the autosome can inform on past selection pressures individuals have been subjected to. Patterns of ROH across the autosome were consistent with estimates of the effective population size; many short ROH were routinely detected in breeds estimated to have a smaller effective population size whereas long ROH, indicative of recent inbreeding were less frequently found across all breeds. Several signatures of selection were also successfully identified, although further annotation is needed to deduce the functions of the uncharacterised genes within these regions. Despite this, the regions identified as under selection in the current study provide an insight into the mechanisms leading to breed differentiation and variation in meat production.

## Supporting information

S1 Fig Principal component analysis for all 6 breeds. (TIF)

S2 Fig Cross validation error values for the admixture results. K is the number of inferred ancestral populations.
(TIF)

S3 Fig The mean sum of runs of homozygosity (ROH) per animal estimated within four different generation categories. ROH were mapped according to their genetic positions (i.e. linkage map positions). ROH length (l cM) within each category was determined using 100/2 *g*, replacing g with the number of generations of interest.
(TIF)

S4 Fig The percentage of chromosome residing in runs of homozygosity (ROH) per breed. (TIF)

S5 Fig The frequency of a single nucleotide polymorphism (SNP) in a run of homozygosity (ROH) for each breed. A) Belclare B) Beltex C) Charollais D) Suffolk E) Texel and V) Vendeen.
(TIF)

S6 Fig The frequency of a single nucleotide polymorphism (SNP) in a run of homozygosity (ROH) estimated across all breeds versus recombination rate. Recombination rate (cM/Mb) was estimated every 500kb. Recombination rate is the solid red line and the occurrence of a SNP in a ROH is the blue dots. A) OAR2 B) OAR4 C) OAR5 D) OAR17 and E) OAR22.
(TIF)

S7 Fig Correlation between global F_ST_ values and hapFLK p-values versus the frequency of a single nucleotide polymorphism (SNP) in a run of homozygosity (ROH). (TIF)

S8 Fig Pairwise F_ST_ values between 9 different breed combinations. A) Belclare versus Beltex B) Belclare versus Charollais C) Belclare versus Suffolk D) Belclare versus Texel E) Belclare versus Vendeen F) Beltex versus Charollais G) Beltex versus Suffolk H) Beltex versus Texel and I) Beltex versus Vendeen. The mean genomic F_ST_ across all SNPs for each pairwise combination is shown in each sub-figure.
(TIF)

S9 Fig Pairwise F_ST_ values between 6 different breed combinations. A) Charollais versus Suffolk B) Charollais versus Texel C) Charollais versus Vendeen D) Suffolk versus Texel E) Suffolk versus Vendeen and F) Texel versus Vendeen. The mean genomic F_ST_ across all SNPs for each pairwise combination is shown on each sub-figure.
(TIF)

S1 Table Runs of homozygosity hotspots within each breed, as defined as the top 1% of SNPs that occurred in a ROH. (DOCX)
