# Supplementary figures and images for: The distribution of runs of homozygosity and selection signatures in six commercial meat sheep breeds
Source: PLoS One. 2017 May 2;12(5):e0176780. doi: 10.1371/journal.pone.0176780 (PMC5413029; doi:10.1371/journal.pone.0176780)

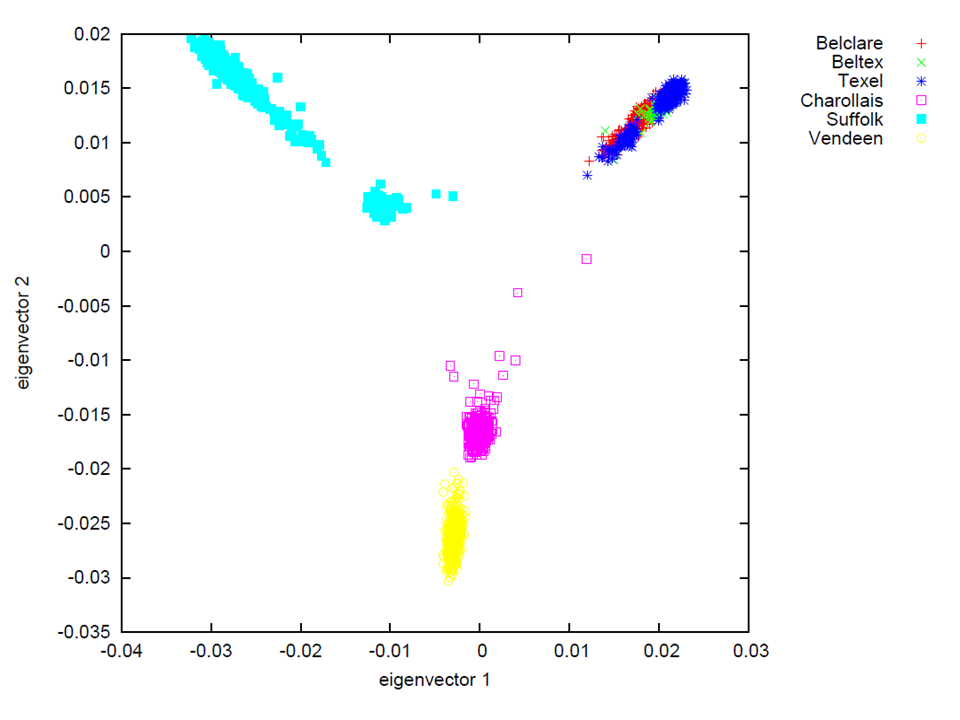

Supplement: S1 Fig — (TIF) [file pone.0176780.s001.tif]

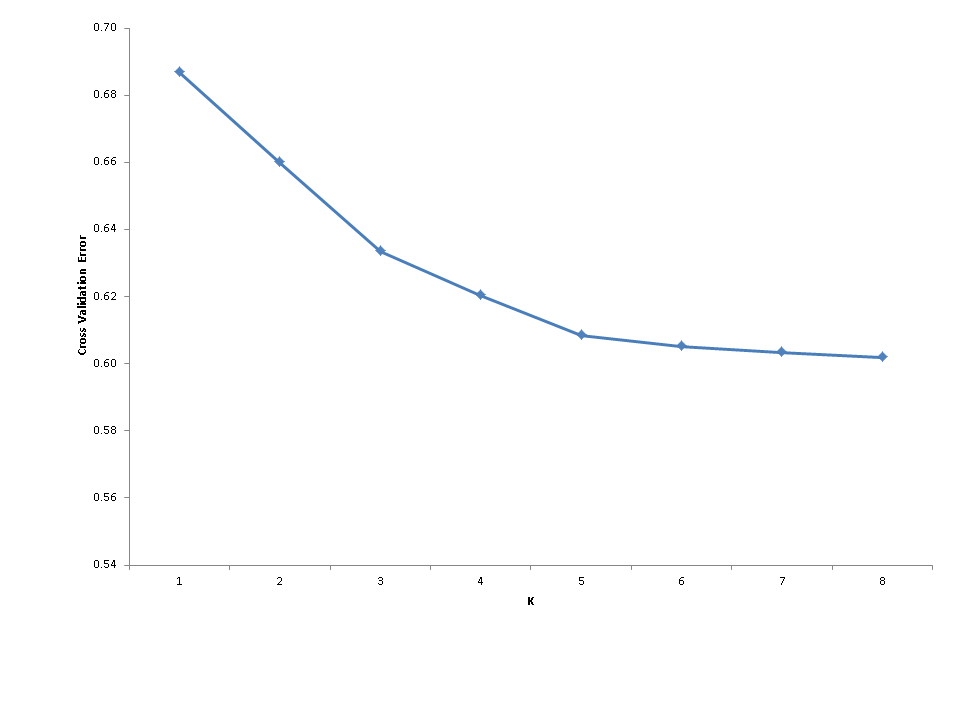

Supplement: S2 Fig — K is the number of inferred ancestral populations. (TIF) [file pone.0176780.s002.tif]

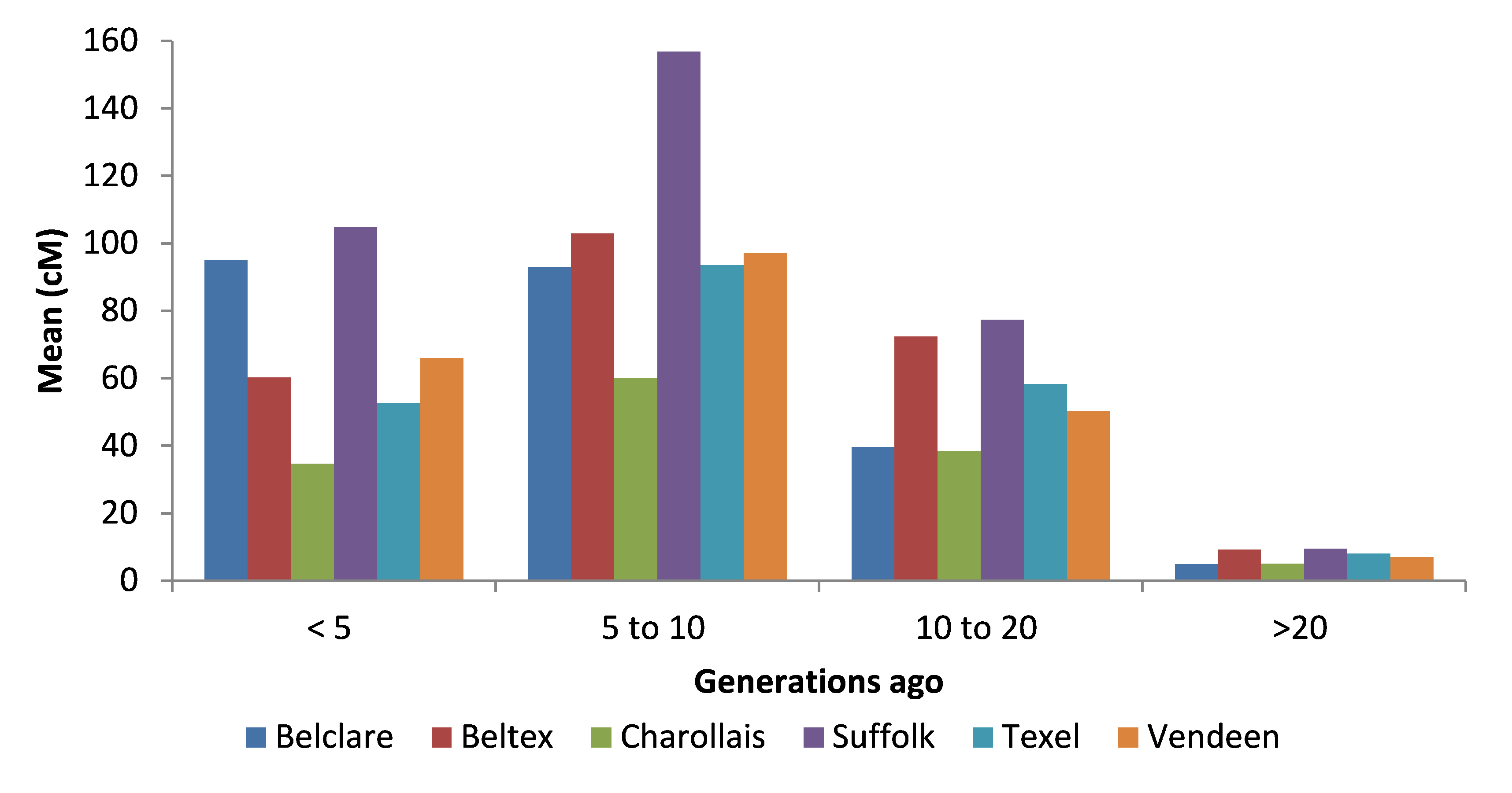

Supplement: S3 Fig — ROH were mapped according to their genetic positions (i.e. linkage map positions). ROH length (l cM) within each category was determined using 100/2 g, replacing g with the number of generations of interest. (TIF) [file pone.0176780.s003.tif]

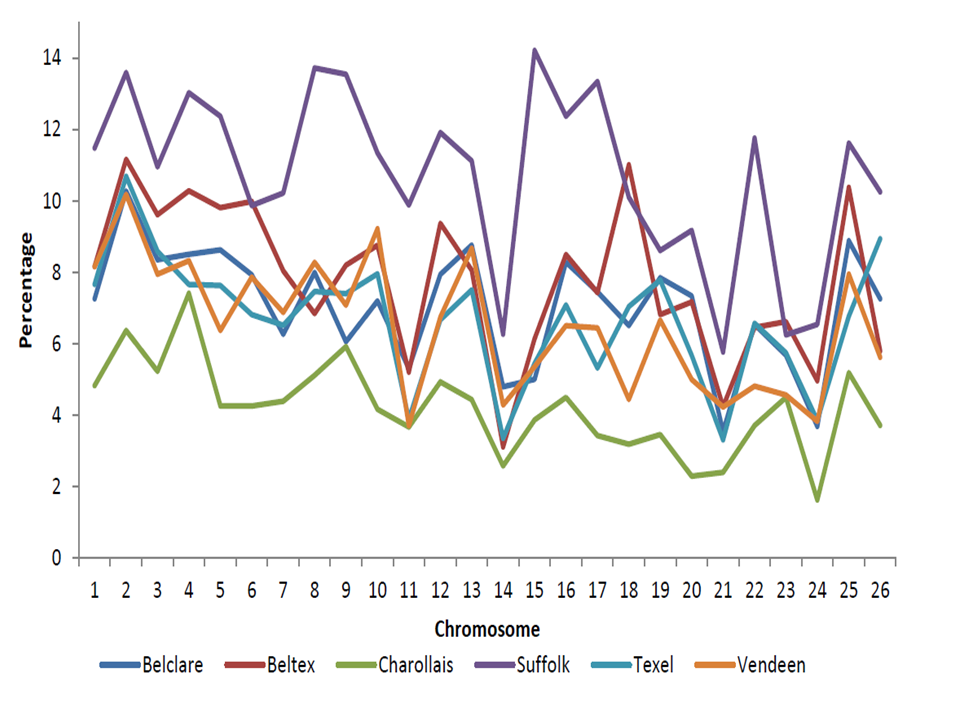

Supplement: S4 Fig — (TIF) [file pone.0176780.s004.tif]

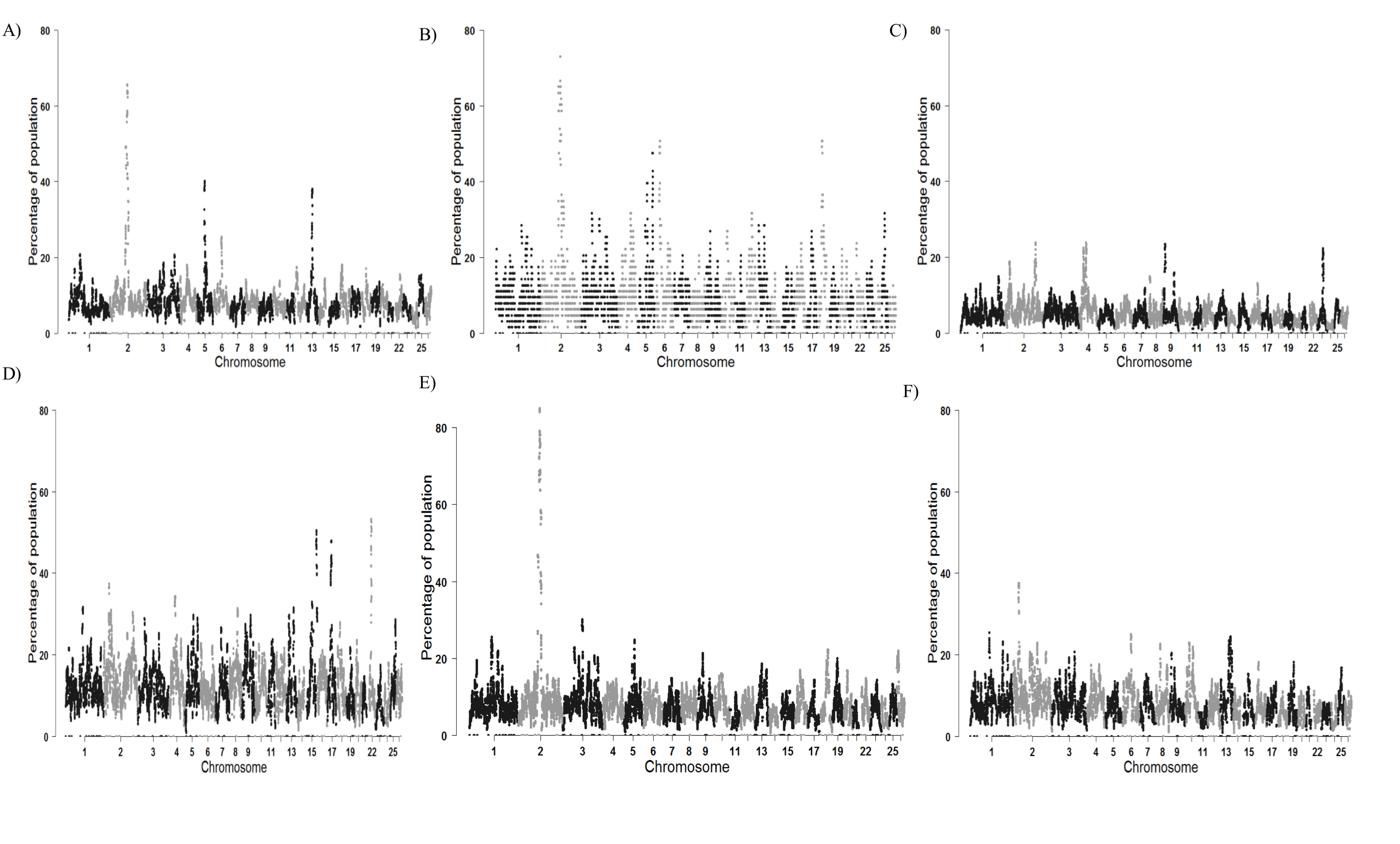

Supplement: S5 Fig — A) Belclare B) Beltex C) Charollais D) Suffolk E) Texel and V) Vendeen. (TIF) [file pone.0176780.s005.tif]

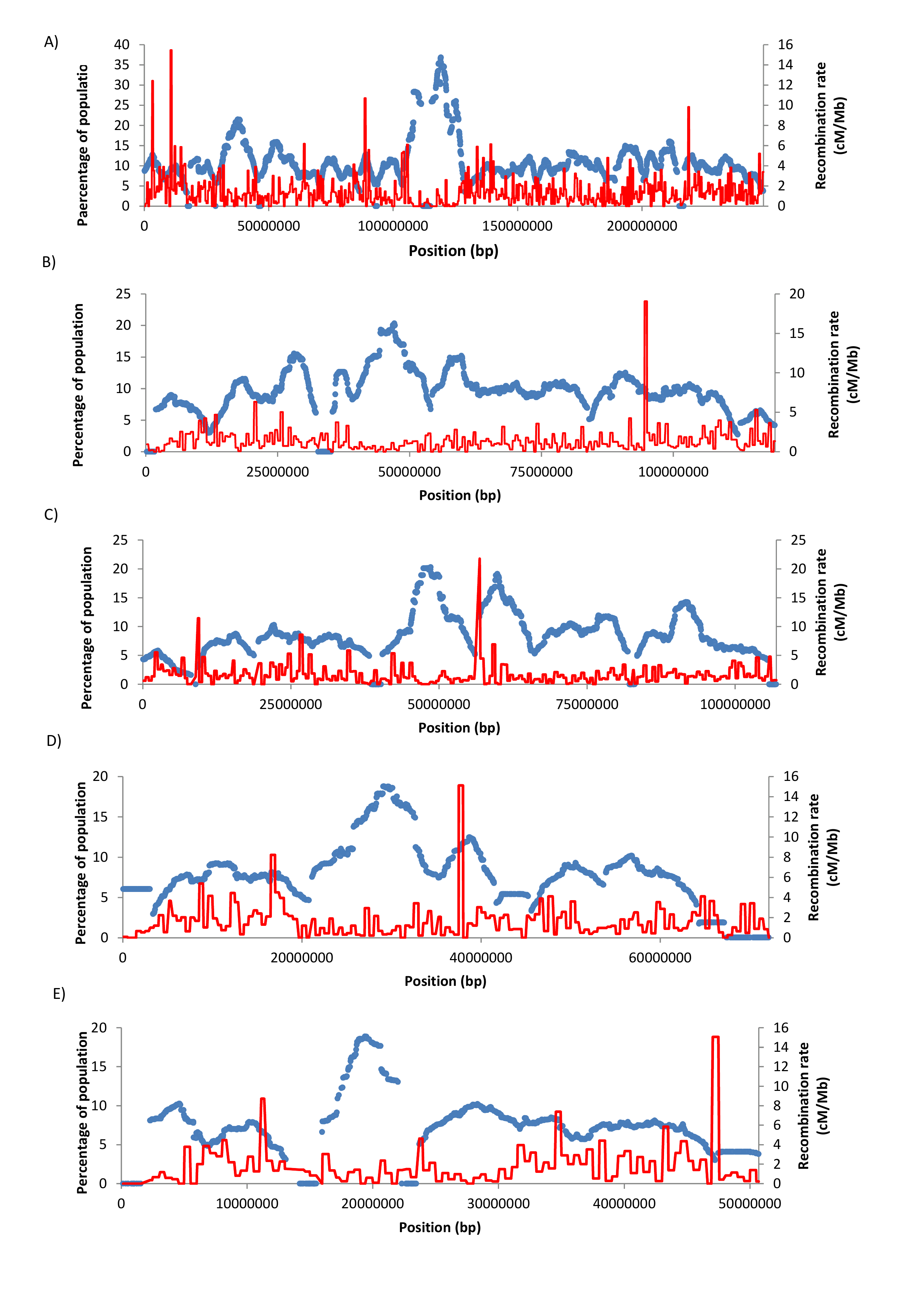

Supplement: S6 Fig — Recombination rate (cM/Mb) was estimated every 500kb. Recombination rate is the solid red line and the occurrence of a SNP in a ROH is the blue dots. A) OAR2 B) OAR4 C) OAR5 D) OAR17 and E) OAR22. (TIF) [file pone.0176780.s006.tif]

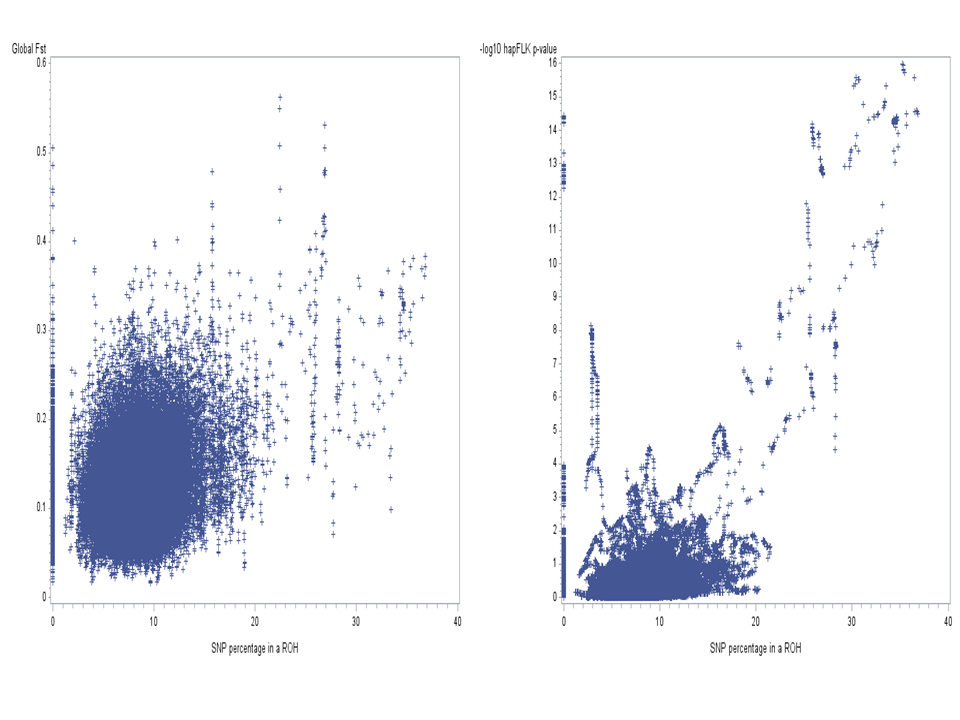

Supplement: S7 Fig — (TIF) [file pone.0176780.s007.tif]

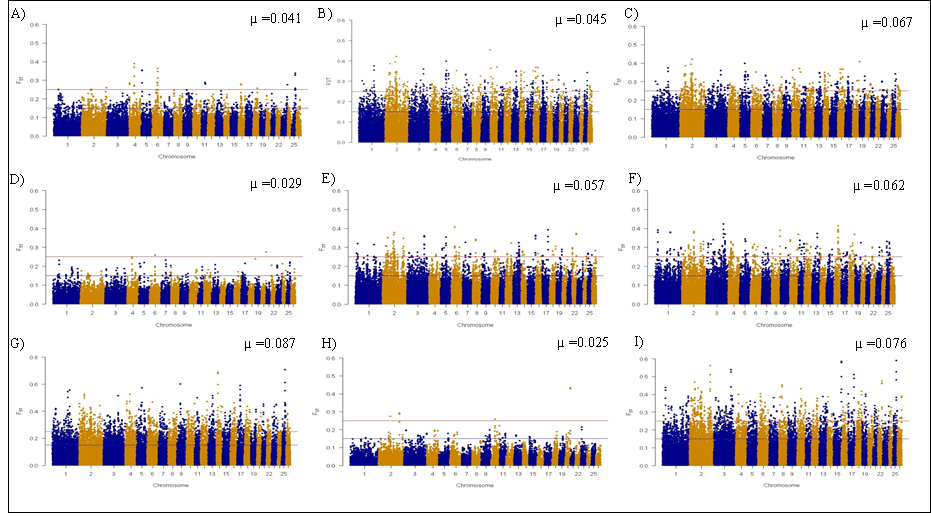

Supplement: S8 Fig — A) Belclare versus Beltex B) Belclare versus Charollais C) Belclare versus Suffolk D) Belclare versus Texel E) Belclare versus Vendeen F) Beltex versus Charollais G) Beltex versus Suffolk H) Beltex versus Texel and I) Beltex versus Vendeen. The mean genomic FST across all SNPs for each pairwise combination is shown in each sub-figure. (TIF) [file pone.0176780.s008.tif]

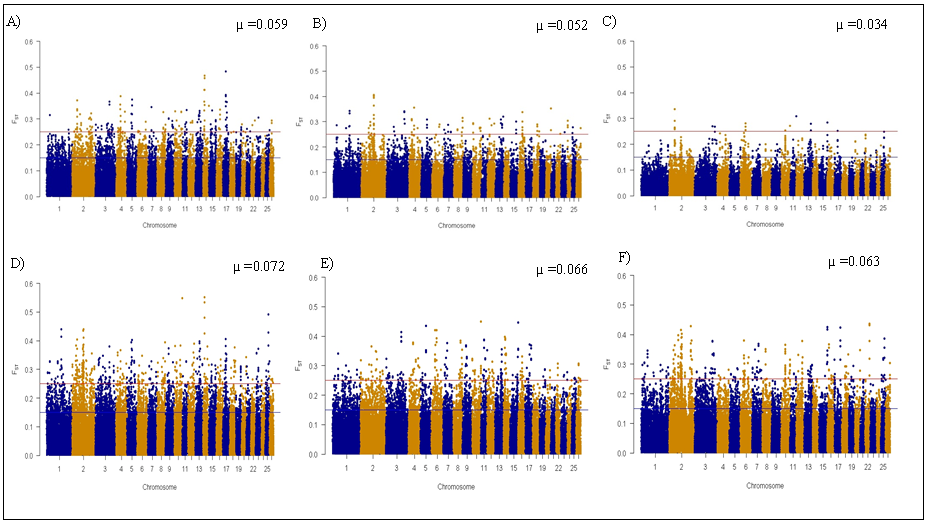

Supplement: S9 Fig — A) Charollais versus Suffolk B) Charollais versus Texel C) Charollais versus Vendeen D) Suffolk versus Texel E) Suffolk versus Vendeen and F) Texel versus Vendeen. The mean genomic FST across all SNPs for each pairwise combination is shown on each sub-figure. (TIF) [file pone.0176780.s009.tif]
