# Supplementary material for: The distribution of runs of homozygosity and selection signatures in six commercial meat sheep breeds
Source: PLoS One. 2017 May 2;12(5):e0176780. doi: 10.1371/journal.pone.0176780 (PMC5413029; doi:10.1371/journal.pone.0176780)
Supplement: S1 Table — (DOCX) [file pone.0176780.s010.docx]

S1 Table. Runs of homozygosity hotspots identified within each breed. The number of significant (sig) SNPs within the top 1% are listed, the average recombination rate (cM/Mb) per hotspot, as well as the likely candidate gene under putative selection within that region.

| Breed | OAR | Position (Mb) | No Sig SNP | Number of genes | Average cM/Mb per hotspot | Candidate genes | Gene function |
| --- | --- | --- | --- | --- | --- | --- | --- |
|  |  |  |  |  |  |  |  |
| Belclare | 2 | 106.98-107.08 | 3 | 1 | 0.14 | [GALNTL6](https://www.ncbi.nlm.nih.gov/entrez/query.fcgi?db=gene&cmd=retrieve&dopt=full_report&list_uids=101121150) | - |
|  | 2 | 107.81-111.34 | 69 | 16 | 0.31 | - | - |
|  | 2 | 115.48-127.39 | 208 | 77 | 0.47 | MSTN | Muscle differentiation |
|  | 5 | 46.37-50.70 | 67 | 107 | 0.73 | EGR1 | Fertility |
|  | 13 | 36.55-41.07 | 73 | 51 | 1.11 | - | - |
|  |  |  |  |  |  |  |  |
| Beltex | 2 | 108.26-111.33 | 60 | 16 | 0.30 | - | - |
|  | 2 | 115.48-126.34 | 207 | 72 | 0.38 | MSTN | Muscle differentiation |
|  | 5 | 58.91-58.91 | 1 | 1 | - | [CSF1R](https://www.ncbi.nlm.nih.gov/entrez/query.fcgi?db=gene&cmd=retrieve&dopt=full_report&list_uids=101109969) | Immunity |
|  | 5 | 89.89-92.05 | 44 | 11 | 1.59 | - | - |
|  | 5 | 92.71-92.98 | 8 | 1 | 0.62 | - | - |
|  | 6 | 26.11-29.34 | 65 | 4 | 1.61 | - | - |
|  | 18 | 22.30-25.14 | 37 | 26 | 0.86 | - | - |
|  |  |  |  |  |  |  |  |
| Charollais | 2 | 32.30-34.52 | 36 | 11 | 1.05 | [NTRK2](https://www.ncbi.nlm.nih.gov/entrez/query.fcgi?db=gene&cmd=retrieve&dopt=full_report&list_uids=101116015) | Fertility |
|  | 2 | 35.56-35.82 | 7 | 3 | 0.82 | - | - |
|  | 2 | 193.29-199.45 | 106 | 34 | 1.62 | HECW2,[STK17B](https://www.ncbi.nlm.nih.gov/entrez/query.fcgi?db=gene&cmd=retrieve&dopt=full_report&list_uids=101119535) | Fertility |
|  | 4 | 26.41-26.73 | 6 | 3 | 1.05 | - | - |
|  | 4 | 27.43-29.78 | 49 | 11 | 1.63 | [ITGB8](https://www.ncbi.nlm.nih.gov/entrez/query.fcgi?db=gene&cmd=retrieve&dopt=full_report&list_uids=101107517) | Fertility |
|  | 4 | 42.84-47.25 | 75 | 38 | 0.47 | RELN | Nematode resistance |
|  | 9 | 81.18-12.68 | 73 | 6 | 0.32 | - | - |
|  | 23 | 33.68-37.89 | 70 | 70 | 1.34 | - | - |
|  |  |  |  |  |  |  |  |
| Suffolk | 1 | 124.18-124.18 | 1 | 0 | - | - | - |
|  | 2 | 38.24-39.68 | 22 | 13 | 0.83 | EBF2 | Adipocyte development |
|  | 4 | 44.49-48.95 | 79 | 38 | 0.49 | RELN | Nematode resistance |
|  | 13 | 54.31-54.32 | 2 | 1 | 0 | - | - |
|  | 15 | 41.33-43.53 | 36 | 34 | 0.55 | - | - |
|  | 15 | 72.40-76.65 | 71 | 74 | 2.96 | - | - |
|  | 17 | 25.77-32.65 | 125 | 30 | 0.95 | - | - |
|  | 22 | 17.51-22.00 | 86 | 88 | 0.87 | - | - |
|  |  |  |  |  |  |  |  |
| Texel | 1 | 122.59-122.72 | 3 | 1 | 3.33 | - | - |
|  | 1 | 123.21-123.21 | 1 | 0 | 1.84 | - | - |
|  | 1 | 123.94-124.80 | 20 | 4 | 1.39 | - | - |
|  | 1 | 126.41-126.49 | 4 | 0 | 1.91 | - | - |
|  | 2 | 108.02-111.33 | 64 | 0 | 0.28 | - | - |
|  | 2 | 115.48-127.31 | 223 | 77 | 0.47 | GDF8 | Muscle differentiation |
|  | 3 | 107.89-112.29 | 95 | 24 | 0.40 | TRHDE | Growth |
|  | 5 | 59.53-60.19 | 12 | 14 | 0.13 | GPX3 | Nematode resistance |
|  |  |  |  |  |  |  |  |
| Vendeen | 1 | 120.79-123.18 | 47 | 26 | 0.62 | - | - |
|  | 1 | 208.53-209.20 | 16 | 1 | 0.59 | - | - |
|  | 2 | 33.79-38.96 | 93 | 71 | 0.81 | NTRK2 | Fertility |
|  | 2 | 154.02-154.80 | 15 | 2 | 0 | - | - |
|  | 6 | 55.96-59.69 | 68 | 32 | 1.22 | - | - |
|  | 8 | 26.89-29.29 | 52 | 26 | 1.25 | FOXO3 | Longevity |
|  | 10 | 29.20-30.56 | 21 | 11 | 1.03 | [RXFP2](https://www.ncbi.nlm.nih.gov/entrez/query.fcgi?db=gene&cmd=retrieve&dopt=full_report&list_uids=101111032) | Horn development |
|  | 10 | 52.67-53.26 | 11 | 2 | 1.16 | - | - |
|  | 13 | 55.92-56.11 | 6 | 3 | 0.95 | - | - |
|  | 13 | 56.35-57.34 | 4 | 23 | 1.68 | EDN3 | Pigmentation |
|  | 13 | 57.39-58.45 | 18 | 11 | 0.83 | PCK1 | Weight gain |
|  | 13 | 63.45-67.80 | 71 | 83 | 2.15 | ASIP | Pigmentation |
